# Prognostic analysis of radiation-induced liver damage following carbon-ion radiotherapy for hepatocellular carcinoma

**DOI:** 10.1186/s13014-024-02444-3

**Published:** 2024-04-22

**Authors:** Kazuhiko Hayashi, Osamu Suzuki, Yushi Wakisaka, Koji Ichise, Hirofumi Uchida, Makoto Anzai, Azusa Hasegawa, Yuji Seo, Shinichi Shimizu, Takayoshi Ishii, Teruki Teshima, Jiro Fujimoto, Kazuhiko Ogawa

**Affiliations:** 1 Department of Radiology, Osaka Heavy Ion Therapy Center, Osaka, Japan; 2 Department of Radiation Oncology, Osaka University Graduate School of Medicine, 2-2 (D10) Yamada-Oka, Suita, Osaka Japan; 3 Department of Radiation Technology, Osaka Heavy Ion Therapy Center, Osaka, Japan

**Keywords:** Hepatocellular carcinoma, Carbon-ion radiotherapy, Radiation-induced liver damage, Prognostic analysis, Child–Pugh score

## Abstract

**Background:**

Radiation-induced liver damage (RILD) occasionally occurs following carbon-ion radiotherapy (CIRT) for liver tumors, such as hepatocellular carcinoma (HCC), in patients with impaired liver function disease. However, the associated risk factors remain unknown. The present study aimed to determine the risk factors of RILD after CIRT.

**Methods:**

We retrospectively analyzed 108 patients with HCC treated with CIRT at the Osaka Heavy Ion Therapy Center between December 2018 and December 2022. RILD was defined as a worsening of two or more points in the Child–Pugh score within 12 months following CIRT. The median age of the patients was 76 years (range 47–95 years), and the median tumor diameter was 41 mm (range 5–160 mm). Based on the pretreatment liver function, 98 and 10 patients were categorized as Child–Pugh class A and B, respectively. We analyzed patients who received a radiation dose of 60 Gy (relative biological effectiveness [RBE]) in four fractions. The median follow-up period was 9.7 months (range 2.3–41.1 months), and RILD was observed in 11 patients (10.1%).

**Results:**

Multivariate analysis showed that pretreatment Child–Pugh score B (*p* = 0.003, hazard ratio [HR] = 6.90) and normal liver volume spared from < 30 Gy RBE (VS_30_ < 739 cm^3^) (*p* = 0.009, HR = 5.22) were significant risk factors for RILD. The one-year cumulative incidences of RILD stratified by Child–Pugh class A or B and VS_30_ < 739 cm^3^ or ≥ 739 cm^3^ were 10.3% or 51.8% and 39.6% or 9.2%, respectively.

**Conclusion:**

In conclusion, the pretreatment Child–Pugh score and VS_30_ of the liver are significant risk factors for RILD following CIRT for HCC.

**Supplementary Information:**

The online version contains supplementary material available at 10.1186/s13014-024-02444-3.

## Background

External beam radiotherapy (EBRT) and stereotactic body radiotherapy (SBRT) are viable treatment options for patients with unresectable or inoperable hepatocellular carcinoma (HCC) [1]. Current guidelines suggest that SBRT is an advanced EBRT technique that delivers high ablative radiation doses, and growing evidence supports the usefulness of SBRT for patients with unresectable, locally advanced, or recurrent HCC [1]. A recent systematic review indicated that SBRT is effective for small HCCs, with a pooled three-year local control rate of 91.0% [2]. Furthermore, the incidence rates of grade 3 or higher hepatic adverse events and radiation-induced liver damage (RILD) were 4.0% and 14.7%, respectively, indicating that the safety of SBRT has been reasonably established. Shen et al. treated medium-sized HCC (median 5.3 cm, 3–7.9 cm) with SBRT and reported a three-year local control rate of 73.3%; RILD was observed in 19.6% of the patients [3]. However, the safety of SBRT for large HCCs remains unclear, as the incidence and severity of RILD may increase with tumor size.

Carbon-ion radiotherapy (CIRT) is a high-dose energy-transfer radiotherapy characterized by excellent dose localization. The number of facilities offering CIRT in Europe and Asia is gradually increasing [4]. CIRT offers better dose concentration than SBRT; consequently, the incidence of high-grade toxicity due to exposure to charged particles, including carbon ions, after CIRT is lower than that observed after SBRT [5, 6]. Notably, CIRT exhibits high efficacy and safety in treating both small and large HCC tumors (0.8–12.0 cm) [7–10]. Although the incidence of severe hepatic adverse events is low, 9% of patients experiences RILD after CIRT, defined as a worsening of two or more points on the Child–Pugh score [7]. However, owing to the limited number of patients who have undergone CIRT for medium-to-large HCCs, information on the associated hepatic adverse events is lacking.

In Japan, CIRT for HCC tumors of ≥ 4 cm is being covered under the Japanese health insurance system since April 2022, resulting in increased utilization of CIRT for medium-to-large HCCs. Although various risk factors for RILD after SBRT have been reported, none have been identified for RILD after CIRT [11–13]^.^ Therefore, predicting the risk of RILD and its severity when administering CIRT to patients with low liver function and large HCC remains challenging. The present study aimed to retrospectively analyze the risk factors of RILD following CIRT for HCC.

## Methods

### Study design and population

This study was approved by our Institutional Review Board (identification number 230601) and conducted in accordance with the Declaration of Helsinki. All patients provided informed consent for using their personal information for research purposes.

A CIRT protocol for HCC was established when the Osaka Heavy Ion Therapy Center opened in 2018, and CIRT was performed accordingly. The eligibility criteria were as follows: HCC diagnosis by imaging (contrast-enhanced computed tomography [CT] or contrast-enhanced magnetic resonance imaging [MRI]) or biopsy; a performance status of 0–2; T1-4N0M0 according to the International Union Against Cancer Tumor-Node-Metastasis Classification (seventh edition) [14]; liver function between Child–Pugh classes A and B; and indication review by our institutional Cancer Board.

We conducted a retrospective survey of all patients treated with CIRT at a dose of 60 Gy (relative biological effectiveness [RBE]) for HCC in four fractions at our institution between December 2018 and December 2022. Consequently, 117 patients were selected. For cases of recurrence after CIRT, liver function data up to the time of recurrence detection were used for analysis, and data thereafter were excluded because of the addition of other therapies. Patients with a follow-up period of less than two months were excluded. Cases in which the recurrent HCCs in the liver were re-irradiated were also excluded. Finally, 108 patients were included in the study.

### Carbon-ion radiotherapy

Marker implantation was performed percutaneously or transvascularly wherever possible. The patients were fixed using an individually tailored fixation shell (Esform; Engineering System Co., Ltd., Matsumoto, Japan), and four-dimensional CT images were obtained in the supine or prone position. The tumors were contoured as gross tumor volume (GTV) on CT images, using contrast-enhanced CT or MRI as reference. The clinical target volume (CTV) was defined as the GTV plus a 0–5 mm margin. The internal target volume (ITV) was defined as the margin accounting for respiratory migration, based on four-dimensional CT, in addition to CTV. A beam-specific margin was set to compensate for the uncertainty in each direction of irradiation [15]. Specifically, a beam-specific planning target volume (PTV) was created, featuring a 5 mm margin on the ITV on the lateral side of the irradiation direction and a margin of 3.5% plus 1 mm of the beam range on the distal and proximal sides of the ITV. Beam-specific PTVs were used to generate dose distributions for each beam. Finally, the PTV was defined as the ITV plus a 2–5 mm safety margin to evaluate the combined dose distribution for each beam [16].

A prescribed dose of 60 Gy (RBE) in four fractions was selected for this study. Basic dose constraints were as follows: the minimum doses delivered to 0.1 cc of the most irradiated gastrointestinal tract volumes (D_0.1 cc_) < 15 Gy (RBE) in four fractions; the skin dose < 50% of the prescribed dose; and normal liver volume was defined as liver volume excluding GTV and the volume of normal liver receiving < 20 Gy (RBE) > 600 cm^3^. All doses were administered four times in one week. The total dose was applied to the isocenter, and the PTV was enclosed conformally at a minimum by the 95.0% isodose line with the prescribed dose. Treatment planning was performed using RayStation (RaySearch Laboratories, Stockholm, Sweden) and VQA Plan (Hitachi Ltd., Tokyo, Japan) [17]. Carbon ions were generated using a heavy ion therapy system (HyBEAT; Hitachi Ltd.). Irradiation was performed in 2–4 fields with 73.3–430.0 MeV/u faster-raster scanning of carbon ions. Respiratory management was performed during the exhalation phase using a respiratory gating system (AZ-733VI; Anzai Medical Co. Ltd., Tokyo, Japan).

After 1 month of treatment, patients were examined. If no abnormalities were observed, enhanced CT or MRI, blood tests, and medical examinations were performed every 2–3 months thereafter.

### RILD analyses

RILD was defined as a worsening of two or more points in the Child–Pugh score within 12 months after CIRT at any time point. Patients with worsening liver function owing to recurrent HCC were excluded. First, patients were divided into two groups: with or without RILD, and univariate analysis of the clinical factors of patients was performed using Fisher’s exact test. Second, the optimal cutoff values for the dosimetric parameters (PTV, mean liver dose, and normal liver volume spared from less than × Gy [RBE] [VS_x_; x = 5, 10, 15, 20, 30, 40, and 50]) and the percentage of normal liver volume receiving equal to or more than × Gy (RBE) (V_x_; x = 5, 10, 15, 20, 30, 40, and 50) were determined using a receiver operating characteristic (ROC) analysis. Third, univariate analysis was conducted on dosimetric parameters, which were divided into two groups using cutoff values and assessed using Fisher’s exact test. Finally, to account for the potential influence of multiple factors, a multivariate analysis was conducted using the Cox proportional hazards model. This analysis included the two most significant variables identified in the initial univariate analysis of clinical factors or dosimetric parameters. We opted for this approach because including too many variables in a multivariate analysis can decrease the model's reliability. A two-tailed *p* < 0.05 was considered statistically significant. All statistical analyses were conducted using the JMP statistical software (version 17.0; SAS Institute Inc., Cary, NC, USA).

## Results

Table 1 summarizes the patient and tumor characteristics. Regarding history of treatment, 72 initial cases and 36 recurrent or residual cases were treated for the present lesion. Of the 36 cases, the details of previous treatment were as follows: 23 transcatheter arterial chemo-embolization, 7 surgery, 5 radiofrequency ablation, and 1 percutaneous ethanol injection therapy. The median tumor diameter was 41 mm (5–160 mm). Considering pretreatment liver function, 98 and 10 patients were categorized as Child–Pugh class A and B, respectively. The median follow-up period was 9.7 months (2.3–41.1 months).

**Table 1:** Patient and tumor characteristics (*N* = 108)

Factors	Values
Age	
Median, years (range)	76 (47–95)
Sex	
Male	77
Female	31
ECOG PS	
0	85
1	18
2	5
Clinical stage	
ΙA	15
ΙB	64
ΙΙ	20
ΙΙΙA	9
Vascular invasion	
Vp0	105
Vp1	0
Vp2	2
Vp3	1
Number of lesions irradiated simultaneously	
1	92
2	6
3	7
4	2
5	1
History of treatment for the present lesion	
Initial case	72
Recurrent or residual case	36
Tumor localization	
Central	39
Peripheral	69
Etiology (overlap present)	
HCV	27
Alcohol	25
NASH	23
HBV	10
Others	24
Median follow-up, months (range)	9.7 (2.3–41.1)
Median tumor diameter, mm (range)	41 (5–160)
Child–Pugh score	
Class A (5 points)	71
Class A (6 points)	27
Class B (7 points)	9
Class B (8 points)	1
ALBI Grade	
Grade 1	60
Grade 2	48

*ECOG PS* Eastern Cooperative Oncology Group performance status, *Vp* portal vein invasion, *HCV* hepatitis C virus, *NASH* nonalcoholic steatohepatitis; *HBV* hepatitis B virus, *ALBI* albumin–bilirubin

**Fig. 1:**
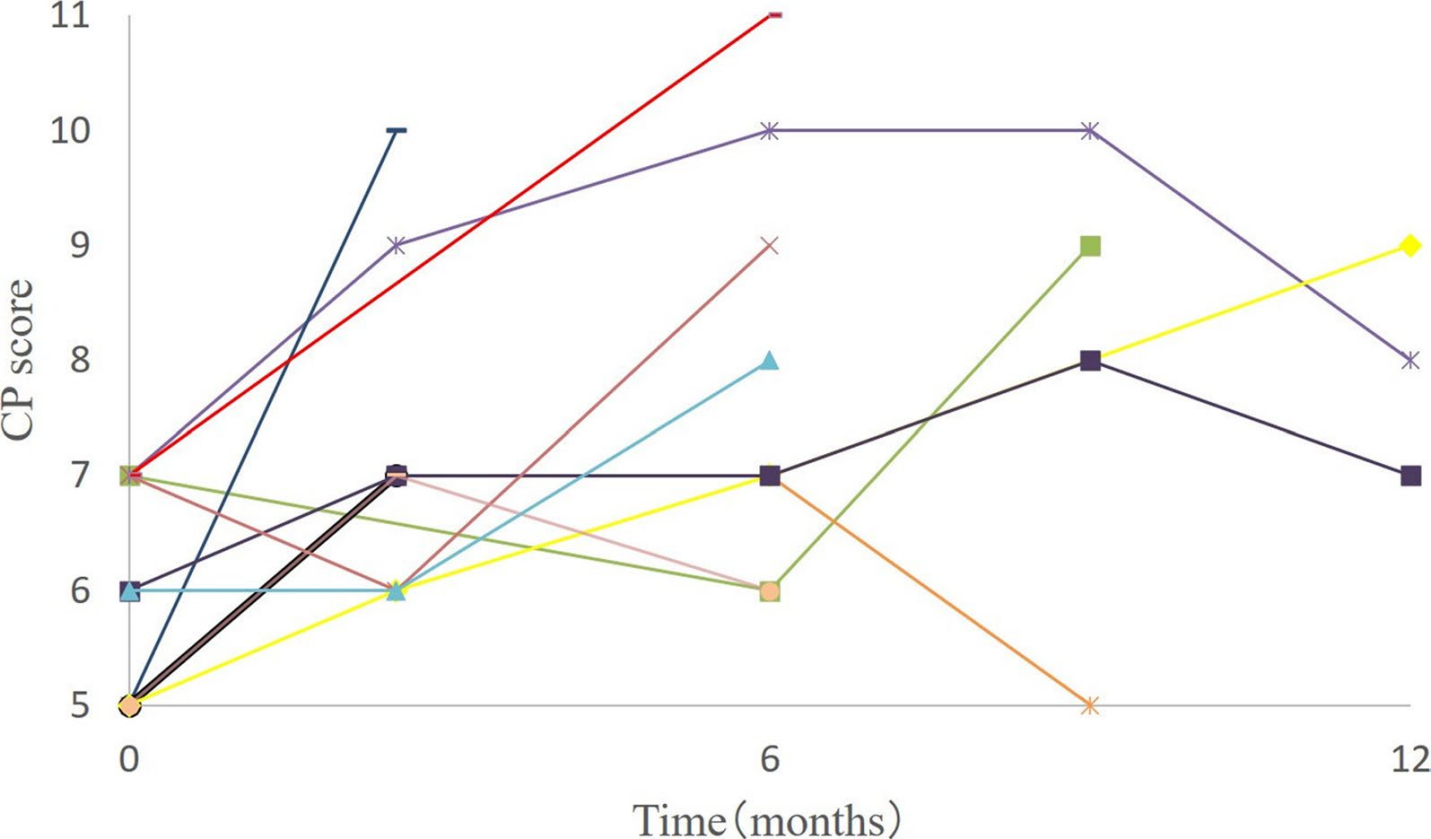
Changes in liver function. Changes in liver function over time in 11 patients with radiation-induced liver damage (RILD) using the Child–Pugh score

Eleven patients developed RILD, characterized by a worsening of two or more points in the Child–Pugh score following CIRT (Additional file 1: Table S1). Of the 11 patients with RILD, 5 were judged as having RILD at 3 months after treatment, 4 at 6 months, and 2 at 9 months. Changes in liver function over time after CIRT were assessed using the Child–Pugh score in 11 patients with RILD (Fig. 1). Liver function improved in 4 of the 11 patients, while it remained deteriorated in the remaining 7 patients. In Child–Pugh classification A, three of the seven (43%) patients improved after RILD, whereas in Child–Pugh classification B, only one of the four (25%) patients improved after RILD.

The results of the univariate analysis of patient and tumor characteristics showed that the pretreatment Child–Pugh score and albumin–bilirubin (ALBI) Grade were significant risk factors for RILD (Table 2). Univariate analysis of dosimetric parameters associated with RILD using the optimal cutoff values determined in the ROC analysis revealed that VS_5_, VS_10_, VS_15_, VS_20_, VS_30_, VS_40_, and VS_50_ were significant risk factors (Table 3). Using two of the most significant variables from the univariate analysis of the clinical factors or dosimetric parameters, multivariate analysis revealed that pretreatment Child–Pugh class B (hazard ratio = 6.90; *p* = 0.003) and VS_30_ < 739 cm^3^ (hazard ratio = 5.22; *p* = 0.009) were significant risk factors for RILD (Table 4).

**Table 2:** Univariate analysis of patient characteristics associated with radiation-induced liver Damage (RILD)

Parameters	Patients with RILD	Patients without RILD	*p*-value
Age			0.759
<76 years	6	47	
≥76	5	50	
Sex			0.172
Male	10	67	
Female	1	30	
ECOG PS			1.00
0	9	77	
1 or 2	2	20	
Number of lesions irradiated simultaneously			0.057
1	7	85	
≥2	4	12	
Etiology			0.153
HCV	2	22	
Alcohol	6	15	
NASH	2	21	
HBV	1	8	
Others	0	31	
Tumor localization			0.200
Central	6	33	
Peripheral	5	64	
Tumor diameter			0.347
<40 mm	3	43	
≥40 mm	8	54	
Child–Pugh score			0.009
Class A	7	91	
Class B	4	6	
ALBI grade			0.011
Grade 1	2	58	
Grade 2	9	39	

*ECOG PS* Eastern Cooperative Oncology Group performance status, *HCV* hepatitis C virus, *NASH* nonalcoholic steatohepatitis, *HBV* Hepatitis B virus, *ALBI* albumin–bilirubin

**Table 3:** Univariate analysis of dosimetric parameters associated with radiation-induced liver damage (RILD)

Variables	Patients with RILD	Patients without RILD	*p*-value
PTV (cm^3^)			0.282
≥58.9	10	70	
<58.9	1	27	
MLD (Gy(RBE))			0.282
≥7.1	10	70	
<7.1	1	27	
VS_5_ (cm^3^)			0.097
≥637	5	69	
<637	6	28	
VS_10_ (cm^3^)			0.048
≥506	8	91	
<506	3	6	
VS_15_ (cm^3^)			0.138
≥697	6	75	
<697	5	22	
VS_20_ (cm^3^)			0.123
≥714	6	77	
<714	5	20	
VS_30_ (cm^3^)			0.036
≥739	6	81	
<739	5	16	
VS_40_ (cm^3^)			0.118
≥937	4	60	
<937	7	37	
VS_50_ (cm^3^)			
≥968	4	61	
<968	7	36	0.110
V_5_ (%)			0.168
≥23.5	10	64	
<23.5	1	33	
V_10_ (%)			0.099
≥19.7	10	63	
<19.7	1	34	
V_15_ (%)			0.284
≥15.0	10	68	
<15.0	1	29	
V_20_ (%)			0.507
≥12.9	9	68	
<12.9	2	29	
V_30_ (%)			
≥13.0	7	50	
<13.0	4	47	0.534
V_40_ (%)			0.534
≥10.8	7	50	
<10.8	4	47	
V_50_ (%)			0.540
≥8.4	7	51	
<8.4	4	46	

*PTV* planning target volume, *MLD* mean liver dose, *RBE* relative biological effectiveness, *VS*_*x*_, normal liver volume spared from less than × Gy (RBE), *V*_*x*_, percentage of normal liver volume receiving equal to or more than × Gy (RBE)

**Table 4:** Multivariate analysis of risk factors for radiation-induced liver damage (RILD)

Factor	Hazard ratio (95% CI)	*p*-value
Child–Pugh (class B vs A)	6.90 (1.93–24.6)	0.003
VS_30_ (< 739 cm^3^ vs ≥ 739 cm^3^)	5.22 (1.52–17.91)	0.009

*VS*_x_ normal liver volume spared from less than × Gy(RBE), *CI* confidence interval

**Fig. 2:**
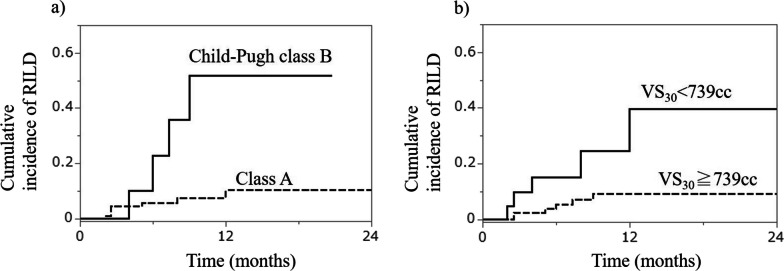
Cumulative incidence of RILD. Cumulative incidence of radiation-induced liver damage (RILD) using the Kaplan–Meier method according to the **a** Child–Pugh score and **b** VS_30_

The cumulative incidence of RILD using the Kaplan–Meier method based on the Child–Pugh score and VS_30_ is shown in Fig. 2a and b, respectively. The one-year incidences of RILD stratified by Child–Pugh class A or B and VS_30_ < 739 cm^3^ or ≥ 739 cm^3^ were 10.3% or 51.8% and 39.6% or 9.2%, respectively.

## Discussion

CIRT presents a promising treatment option for HCC, particularly for patients with unresectable HCC [7–10]. In Japan, CIRT is already covered by the Japanese health insurance system for HCCs larger than 4 cm. To date, no study has predicted RILD after CIRT, and, therefore, estimating the risk of RILD or preventing serious RILD has been challenging. To the best of our knowledge, this is the first study to determine pretreatment Child–Pugh score B and VS_30_ < 739 cm^3^ as risk factors for RILD due to CIRT. Moreover, our study showed that the one-year cumulative incidences of RILD stratified by Child–Pugh class A or B and VS_30_ < 739 cm^3^ or ≥ 739 cm^3^ were 10.3% or 51.8% and 39.6% or 9.2%, respectively. Our findings demonstrate that adhering to the dose constraint of VS_30_ ≥ 739 cm^3^ can effectively prevent RILD, providing valuable insights into planning CIRT treatment.

Although there are no reports on the risk of RILD due to CIRT, there are some reports on the risk of RILD after X-ray SBRT or proton beam radiotherapy. Previous studies on SBRT-induced RILD have reported various risk factors, such as V_15_, VS_10_, pretreatment Child–Pugh score, mean liver dose, high doses to 800 cm^3^ of the liver, and lower platelet count [11–13]. Hsieh et al. evaluated proton radiotherapy-induced RILD and reported GTV, pretreatment Child–Pugh score, and unirradiated liver volume/standard liver volume as significant risk factors [18]. In the present study, the pretreatment Child–Pugh score and VS_30_ of the liver were identified as significant risk factors for RILD after CIRT. While direct comparisons between CIRT, proton, and X-ray treatments are challenging because of the differences in radiation quality and dose fractionation, the pretreatment Child–Pugh score consistently emerges as a risk factor for RILD. Moreover, a dose constraint of VS_15_ ≥ 700 cm^3^ has been proposed for SBRT according to QUANTEC [18], supporting our finding of VS_30_ ≥ 739 cm^3^.

In the past, RILD due to X-rays was classified into classical and non-classical RILD [19]. Classical RILD, which presents with hepatomegaly, anicteric ascites, and elevated alkaline phosphatase, occurs following whole liver irradiation or conventional RT; however, SBRT-induced RILD belongs to the non-classical category. The symptoms of non-classical RILD vary based on SBRT or proton therapy, with the most common and the primary definition considered in this study being a worsening of the Child–Pugh score by two or more points [19]. However, some studies have included criteria such as hepatobiliary enzymes elevated five times above the upper limit of normal or ascites [12, 18]. These differences arise from the absence of a standardized method with high sensitivity and specificity for detecting non-classical RILDs.

Recovery from RILD has been reported in some cases. Jun et al. reported an improvement in Child–Pugh classification A, but not in B, after SBRT [12]. The present study showed that 43% of patients with pretreatment Child–Pugh classification A showed improvement after RILD, whereas only 25% of patients with classification B showed improvement. These results indicate that recovery from RILD can be difficult; therefore, CIRT must be used to proactively prevent RILD. Although CIRT has shown high local control rates for HCC, new lesions in the liver outside the irradiated field typically recur [7–10]. In such cases, if RILD from CIRT persists, impaired liver function may complicate definitive local treatments (such as surgery, radiofrequency ablation, SBRT, and CIRT) for new HCC lesions. Therefore, our results suggest that the one-year incidence of RILD can be reduced from 39.6% to 9.2% by setting VS_30_ ≥ 739 cm^3^ as a dose constraint in the optimal CIRT treatment planning.

For patients who have undergone treatment for HCC, liver damage can occur for three reasons: HCC recurrence, spontaneous worsening of cirrhosis, and RILD. Patients with HCC recurrence detected after CIRT were excluded from the analysis. In patients with cirrhosis who have maintained Child–Pugh A or B liver function, cirrhosis generally worsens slowly, but RILD occurs within several months of treatment [19]. Therefore, RILD in this study was defined as liver function deterioration within 12 months of CIRT. However, even if the definition of RILD included the time from treatment, spontaneous deterioration of cirrhosis cannot be completely ruled out because both RILD and spontaneous deterioration of cirrhosis are measured using the same Child–Pugh classification of liver damage. Our study has several limitations. First, this was a single-center retrospective study with a small sample size of only 11 cases of RILD. Second, our study included a predefined dose constraint of the volume of the normal liver receiving < 20 Gy (RBE) as > 600 cm^3^, introducing patient and treatment plan bias. Third, it was difficult to accurately distinguish RILD from spontaneous worsening of liver cirrhosis. Fourth, the number of patients with Child–Pugh classification B was only 10.

## Conclusions

In conclusion, our findings showed that the pretreatment Child–Pugh score and VS_30_ of the liver were significant risk factors for RILD after CIRT for HCC, warranting further large-scale prospective trials.

### Supplementary Information

**Additional file 1. Table S1**. Changes in Child–Pugh (CP) scores before and after carbon-ion radiotherapy. **Table S2**. Results of ROC analysis for significant dosimetric parameters associated with radiation-induced liver damage (RILD)

## Data Availability

Not applicable.

## Abbreviations

CIRT: Carbon-ion radiotherapy
RBE: Relative biological effectiveness
LC: Local control
OS: Overall survival
PTV: Planning target volume
MRI: Magnetic resonance imaging
CT: Computed tomography
PET: Positron emission tomography
GTV: Gross tumor volume
RILD: Radiation-induced liver toxicity
CIRT: Carbon-ion radiotherapy
HCC: Hepatocellular carcinoma
ALBI: Albumin–bilirubin
SBRT: Stereotactic body radiotherapy
ROC: Receiver operating characteristic
VSx: Absolute liver volume spared from at less × Gy (RBE) (x = 5, 10, 15, 20, 30, 40, and 50)
Vx: Percentage of normal liver volume-the gross tumor volume receiving equal to or more than × Gy (RBE) (x = 5, 10, 15, 20, 30, 40, and 50)
