# Supplementary material for: Prognostic analysis of radiation-induced liver damage following carbon-ion radiotherapy for hepatocellular carcinoma
Source: Radiat Oncol. 2024 Apr 22;19:51. doi: 10.1186/s13014-024-02444-3 (PMC11034055; doi:10.1186/s13014-024-02444-3)
Supplement: Supplementary file 1 — Additional file 1. Table S1. Changes in Child–Pugh (CP) scores before and after carbon-ion radiotherapy. Table S2. Results of ROC analysis for significant dosimetric parameters associated with radiation-induced liver damage (RILD) [file 13014_2024_2444_MOESM1_ESM.docx]

**Appendix**

**Table S1.** Changes in Child–Pugh (CP) scores before and after carbon-ion radiotherapy.

|  |  | **After treatment, CP score** | | | | | | |
| --- | --- | --- | --- | --- | --- | --- | --- | --- |
| Pretreatment  CP score | Patients | **5** | **6** | **7** | **8** | **9** | **10** | **11** |
| 5 | 71 | 52 | 14 | 3 | 0 | 1 | 1 | 0 |
| 6 | 27 | 5 | 13 | 7 | 2 | 0 | 0 | 0 |
| 7 | 9 | 0 | 2 | 3 | 0 | 2 | 1 | 1 |
| 8 | 1 | 0 | 0 | 0 | 0 | 1 | 0 | 0 |
| Sum | 108 | 57 | 29 | 13 | 2 | 4 | 2 | 1 |

**Table S2.** Results of ROC analysis for the significant dosimetric parameters associated with radiation-induced liver damage (RILD)

| Variables | AUC | Cutoff value (cm^3^) | Sensitivity | Specificity |
| --- | --- | --- | --- | --- |
| VS_10_ | 0.568 | 506 | 0.949 | 0.273 |
| VS_30_ | 0.562 | 739 | 0.835 | 0.455 |

Abbreviations: RBE, relative biological effectiveness: VS_x_, normal liver volume spared from less than x Gy (RBE)
